# Preventing vector-borne diseases at major sport events: Addressing the challenges for FIFA 22 in Qatar

**DOI:** 10.1371/journal.pntd.0009135

**Published:** 2021-03-11

**Authors:** Francis Schaffner, Devendra Bansal, Mohammed Hamad J. Al-Thani, Hamad Al-Romaihi, Elmoubasher Abu Baker Abd Farag

**Affiliations:** 1 Francis Schaffner Consultancy, Riehen, Switzerland; 2 Ministry of Public Health, Doha, Qatar; Vienna, AUSTRIA

The 2022 FIFA World Cup Qatar has necessary requirements for the prevention and preparedness for any potential disease transmission, including vector-borne diseases (VBDs). Hence, we examined the current status of vectors and VBDs of public health importance in Qatar, based on a systematic literature review that complemented a vector control situation and needs assessment performed in November 2017 [1].

**Fig 1:**
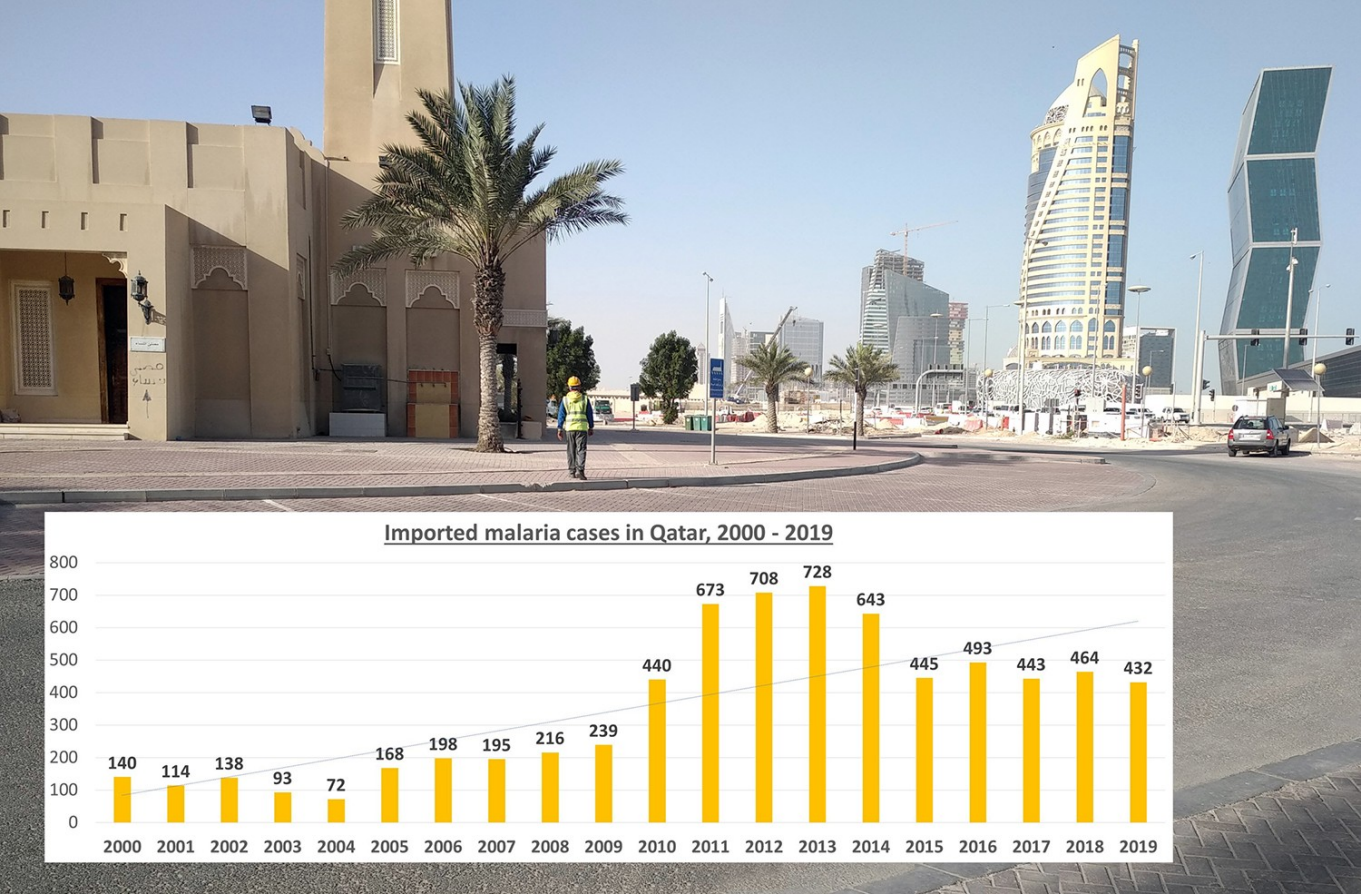
Annual numbers of reported (imported) human malaria cases in Qatar, 2000–2019. Data source: Ministry of Public Health of Qatar; WHO Regional Office for the Eastern Mediterranean. *Photo credit*: *Francis Schaffner*.

The literature reveals that there are no records of locally acquired VBD cases in Qatar, but cases were documented among expatriate workers and travellers who returned from an endemic country. Data on VBD cases remain scarce except for malaria, with a high number of imported cases reported during the last 2 decades (Fig 1) [2]. The presence of native arthropod vectors has not been widely documented to date, with only 30 vector species, including 20 mosquitoes, 2 fleas, 1 louse, 1 fly, and 6 ticks on record. Although no sand flies are described in Qatar, we found reports of locally acquired *Leishmania* infections [3]. This, together with the high numbers of sand fly and other vector species that are reported in neighbouring countries with similar environmental conditions [4], suggests that Qatar may experience more species. Overall, Qatar has some capacities with regard to vector control, but no national plan currently exists, and vector surveillance is still in its infancy.

In Qatar, some structural changes are risk factors to facilitate VBD upsurge and transmission, i.e., (1) a rise in the number of pathogen introduction events in response to a rapid increase of Qatari population (×3.8 over 15 years) due to high number of migrants that represent more than 75% of the population together with a considerable influx of tourists’ (×7.5 over 14 years) [5]; (2) the high proportion (more than 99%) and increase of urban population (×4.7 over 15 years) [5], providing ideal conditions that increase the likelihood of VBD local transmissions [6]; and (3) the booming of freight air transport (×24.6 over 14 years), port traffic (+29% over 14 years), and merchandise import (×10.6 over 14 years) [5], which increases the risk of introduction of vectors, pathogen infected or not [7].

There is a clear need for capacity building in epidemiology and vector entomology, as well as on the organisational level, to mitigate and improve the VBD risk assessment and management. It is essential to define sustainable solutions for VBD control, management, and prevention, and a number of recommendations are suggested (Box 1) to prevent VBD outbreaks during the 2022 FIFA World Cup Qatar or any other major local sport event.

**Box 1. Recommendations for addressing VBD risk in Qatar**

A number of recommendations are suggested in order to (1) strengthen the integrated vector management (including surveillance and control) and (2) ensure effective VBD outbreak preparedness and response, within a “One Health” approach, by complying with WHO resolutions:

- Define a national integrated strategy for the management of VBDs in Qatar, to be developed within a surveillance and control plan for the main VBDs at risk for the country, identify all contributors and their tasks, build a coordination unit, and allocate adequate financial resources.
- Build capacities in entomology to increase knowledge of local vector ecology and competences for vector identification and vector surveillance.
- Build capacities in research infrastructure including in laboratory diagnostic, for both molecular detection of pathogens and identification of vectors.
- Develop coordination and enhance national and international collaborations to build intra- and inter-sectoral networking on vectors and pathogens they transmit.
- Build an integrated surveillance and control system of both vectors and pathogens, based on integrated vector management, with prioritised targets in term of vector species, pathogens, and high-risk areas.
- Develop a national public health pesticide management policy to tackle weaknesses in pesticide use or management and to avoid and solve potential problems of impact on human health and nontarget organisms.
- Plan an evaluation and quality check process to adjust the surveillance system when/where needed.
